# Supplementary material for: Complexome profiling of the Chlamydomonas psb28 mutant reveals TEF5 as an early PSII assembly factor
Source: Plant Cell. 2025 Mar 17;37(6):koaf055. doi: 10.1093/plcell/koaf055 (PMC12164590; doi:10.1093/plcell/koaf055)
Supplement: koaf055_Supplementary_Data [file koaf055_supplementary_data.zip › psb28_interactive.zip!/plots/intensity/qualityPlots/peptides/_Cre06.g278800.t1.2.html]

### Cre06.g278800.t1.2

Trivial name:   
SeqCoverage [median]: 9.30  
Mass: 28.45 kDa  
  
 

| Sequence | MissedCleavage | Mass [Da] | wtIntensity | muIntensity | Score |
| --- | --- | --- | --- | --- | --- |
| AVAPAVAK | 0 | 725.44 | 26148.26 | 38533.29 | 117.70 |
| LQQDWFETIK | 0 | 1306.66 | 659.02 | 0.00 | 73.23 |
| SSSIDVK | 0 | 734.38 | 4153.55 | 11730.57 | 125.01 |
|  |  |  | 30960.8 | 50263.9 |  |
